# Extended-Spectrum-β-Lactamase- and AmpC-Producing *Escherichia coli* in Domestic Dogs: Spread, Characterisation and Associated Risk Factors

**DOI:** 10.3390/antibiotics10101251

**Published:** 2021-10-15

**Authors:** Nicoletta Formenti, Andrea Grassi, Giovanni Parisio, Claudia Romeo, Flavia Guarneri, Laura Birbes, Alessandra Pitozzi, Federico Scali, Antonio Marco Maisano, Maria Beatrice Boniotti, Paolo Pasquali, Giovanni Loris Alborali

**Affiliations:** 1 Istituto Zooprofilattico Sperimentale della Lombardia e dell’Emilia Romagna “Bruno Ubertini”, Via Bianchi 7/9, 25124 Brescia, Italy; andrea.grassi@i-vet.it (A.G.); giovanni.parisio@izsler.it (G.P.); flavia.guarneri@gmail.com (F.G.); laura.birbes@izsler.it (L.B.); alessandra.pitozzi@izsler.it (A.P.); federico.scali@izsler.it (F.S.); antoniomarco.maisano@izsler.it (A.M.M.); mariabeatrice.boniotti@izsler.it (M.B.B.); giovanni.alborali@izsler.it (G.L.A.); 2 Dipartimento di Scienze degli Alimenti e del Farmaco, Università di Parma, Parco Area delle Scienze 27/A, 43124 Parma, Italy; claudia.romeo@yahoo.it; 3 Dipartimento di Sicurezza Alimentare, Nutrizione e Sanità Pubblica Veterinaria, Istituto Superiore di Sanità, Viale Regina Elena 299, 00161 Roma, Italy; paolo.pasquali@iss.it

**Keywords:** extra-urban environments, *bla*_CMY_, cefotaxime/clavulanic acid, ceftazidime/clavulanic acid, *bla*_CTX-M_, cefoxitin, multidrug resistance (MDR), carbapenems

## Abstract

In veterinary medicine, the issue of antimicrobial resistance was mainly addressed in food-producing animals (although companion animals also deserve attention). Indeed, these species may be reservoir of resistant microorganisms, such as extended-spectrum β-lactamase and AmpC (ESBL/AmpC)-producing bacteria. Dogs in particular may transmit them to close-contact humans. Overall 266 faecal samples of healthy dogs were microbiologically and molecularly analyzed to investigate ESBL/AmpC-producing *Escherichia coli* and the effects of host and environmental factors on their spread. A prevalence of 25.9% of ESBL/AmpC-producing *E. coli*, supported by *bla*_CTX-M_ (79.7%), *bla*_TEM_ (47.8%), *bla*_CMY_ (13%), and *bla*_SHV_ (5.8%) gene detection, emerged. Dogs frequenting extra-urban environments showed significantly higher odds of being positive to ESBL/AmpC *E. coli* (30.2%) compared to urban dogs (16.7%) identifying the environment as a risk factor. About 88.4% of isolates were resistant to cephalosporins, 8.7% to cephalosporins and carbapenems, and 2.9% to cephalosporins, carbapenems, and penicillins. ESBL/AmpC-producing *E. coli* expressing *bla*_CMY_ were significantly more resistant to cefoxitin, cefotaxime/clavulanic acid and ceftazidime/clavulanic acid, highlighting its negative effects. Our results suggest the role of domestic dogs as a maintenance host of ESBL/AmpC-producing *E. coli* leading to a constant health monitoring. The recorded resistances to carbapenems implies attention and further investigations.

## 1. Introduction

Antimicrobial resistance (AMR) represents a ‘one health’ problem since involves a wide range of participants, humans, animals, and the environment [1]. The emergence and the rapid dissemination of antibiotic-resistant bacteria poses substantial risks for human health with global deaths related to AMR predicted to reach 10 million by 2050, more than the current mortality associated with different forms of cancer [2]. Among the most important bacteria that contributes to the complexity of AMR, Enterobacteriaceae producing extended spectrum beta-lactamase (ESBL) and plasmid mediated AmpC beta-lactamase (AmpC) emerged as a healthcare problem worldwide in human and veterinary medicine [3] due to their resistances to third and fourth generation cephalosporins and to the majority of β-lactams [4]. These resistant strains can spread both clonally and horizontally among different lineages, even to non-pathogenic bacteria [5], complexly with multiple reservoirs and different transmission routes [3]. For years major attention was given on ESBL/AmpC- producing bacteria in food producing animals [6,7], potentially transmitting to humans through the food chain, although these strains are found also in companion animals [6,8]. In this regard, the related potential zoonotic risk should be emphasized [3] in light of the increased number of people living with pets, mainly dogs, over the last few decades [9]. Indeed, AMR monitoring in companion animals represents a crucial point due to their potential role, especially of dogs, of reservoir of resistant bacteria likely transmitted to humans through frequent or intimate direct or indirect contacts sharing the same environments [9,10,11,12,13]. Although AMR is closely induced by the over-use of antibiotics [14,15], the use of antimicrobials in companion animals has received little attention and remains unregulated unlike the guidelines or legal restrictions active for farm animals in many countries [16]. For example, cephalosporins, whose use in farm animals is restricted or prohibited in some countries, are still widely used in animals despite they are among the critically important classes for use in human medicine [16]. The continued use of cephalosporins/other important molecules in companion animals could induce resistances that may be transmitted to humans. Thus, information on the presence of AMR in the bacterial flora of dogs should be acquired. In particular, attention should be posed to those bacteria well adapted for colonisation of both humans and animals, such as *Escherichia coli*, as shared environments provide the opportunity for rapid dissemination of these strains from one host to the other [10]. In addition, the human population is more likely to be exposed to bacteria present in the feces of dogs (e.g., owners picking up after their dogs have defecated) [17]. Although a few studies reported the increase of resistance to several antimicrobials in companion animal isolates over time [18], AMR scientific data should be broadened and monitored over the years concerning domestic dogs in light of their close contacts with owners and the related potential risks of AMR transmission.

Here, we carried out an epidemiological investigation of ESBL/AmpC β-lactamase producing *Escherichia coli* in domestic dogs with different living attitudes in order to evaluate (i) their prevalence, (ii) their phenotypic and molecular antimicrobial resistances, and (iii) the host and environmental factors influencing the spread of these pathogenic bacteria.

## 2. Results

A total of 69 ESBL/AmpC-producing *E. coli* isolates were microbiologically detected over the overall sampling of 266 (25.9%; 95%CI: 20.6–31.2). The phylogenetic analysis showed the presence of seven *E. coli* phylo-groups. Specifically, most of the isolates belonged to either phylogenetic group B1 (21/69; 30.4%) or A (19/69; 27.5%). Six samples each were assigned to group B2, C, E, F (8.7% each), while group D was the least represented (5/69; 7.2%).

Statistical analysis showed the effect of the type of environment on the probability of testing positive to ESBL/AmpC *E. coli*. In particular, dogs frequenting extra urban environments showed significantly higher odds of being positive (OR = 2.23; 95%CI: 1.10–4.53), with a prevalence of 30.2% (95%CI: 23.6–36.8%), compared to 16.7% (8.2–25.1%) of urban dogs. The age class was not associated with the probability of being positive to ESBL/AmpC *E. coli* (χ^2^_2_ = 2.71; *p* = 0.26). Considering only the subset of individuals (*n* = 189) that frequented extra urban environments, ESBL/AmpC *E. coli* prevalence in hunting dogs (*n* = 131) was 32.8% (95%CI: 24.6–41.0%), while it was 24.1% (95%CI: 12.8–35.5%) in non-hunting dogs (*n* = 58), yielding a non-significant difference (χ^2^_1_ = 1.44; *p* = 0.23).

*Bla*_CTX-M_ was the most detected resistance gene, found in 55 isolates (79.7%) and equally common in all the phylogenetic groups, with a percentage ranging from 66.7% to 100% (Table 1). *Bla*_TEM_ was found in 33 isolates (47.8%) and was significantly more associated with groups A, C and E (Fisher’s exact test: *p* = 0.03). Sequence analysis of the amplicons (*n* = 25, eight cases were non-typeable) revealed the presence of TEM-1 (24/25), TEM-57 (1/25).

*Bla*_CMY_ and *bla*_SHV_ were less common, occurring in nine (13%) and four (5.8%) of the isolates: the latter was indeed detected in groups A and B1. Sequence analysis of the amplicons revealed the presence of SHV-12 (4/4). Neither dogs’ age class nor the type of environment influenced the presence of specific resistance genes isolates (all *p* > 0.5). Regarding the combinations of resistance genes (Table 2), the presence of *bla*_CTX-M_ alone was the most frequently observed pattern in the 69 isolates, followed by a combination of *bla*_CTX-M_ and *bla*_TEM_. In general, in most cases (38/69) a single resistance gene was present, and only in one isolate did we detect more than two genes (Table 2).

All of the 69 ESBL/AmpC *E. coli* isolates were resistant to at least one of the ten antimicrobials (AMs) tested by MIC (median: three; range: two to nine). Most of these (61/69; 88.4%) were resistant to AMs belonging to a single AM class, with only six (8.7%) and two (2.9%) isolates showing resistance to two and all the three AM classes, respectively.

Most of the isolates were resistant to cephalosporins, in particular to FOT (69/69; 100%), FEP (60/69; 86.9%), and TAZ (62/69; 89.8%) (Table 3). Resistance to FOX (14/69; 20.3%) and to F/C and T/C was less frequent (17.4% and 18.8%). Resistance to carbapenemase was rare, with eight isolates resistant to ETP (11.6%), one to MERO (1.4%) and none to IMI. Only two isolates were resistant to TRM (2.9%).

Resistance to cefoxitin and both of the cephalosporins/clavulanic acid antimicrobials was associated to the presence of the *bla*_CMY_, with isolates expressing *bla*_CMY_ being more likely of showing resistance to these antimicrobials (Table 4 and Table 5). Resistance to the other antimicrobials was not related to the presence of any of the examined genes (all *p* > 0.5). None of the genes influenced the number of antimicrobials nor the number of AM classes that isolates were resistant to (all *p* > 0.05). *Bla*_CMY_ influenced the number of antimicrobials an isolate was resistant to: isolates with this gene were resistant on average to 0.47 ± 0.23 SE more antimicrobials than isolates without it (χ^2^_1_ = 4.25; *p* = 0.044). However, none of the genes influenced the number of wider AM classes that isolates were resistant to (all *p* > 0.05).

Resistance to the other antimicrobials was not related to the presence of any of the examined genes (all *p* > 0.5).

## 3. Discussion

The present study revealed a high prevalence of ESBL/AmpC-producing *E. coli* with higher odds of being positive in dogs frequenting extra urban environments, highlighting the role of the environment as a risk factor. Most of ESBL/AmpC-producing *E. coli* were resistant to AMs belonging to cephalosporin class, with six and two isolates showing resistance to two (cephalosporins and carbapenems) and to all the three AM classes (cephalosporins, carbapenems and penicillins), respectively. Resistance to FOX, F/C and T/C was positively associated to the presence of *bla*_CMY_: ESBL/AmpC-producing *E. coli* expressing *bla*_CMY_ were more likely to show resistance to these antimicrobials.

The most frequent ESBL/AmpC-producing *E. coli* phylogenetic group was B1 (21/69; 30.4%), followed by A (19/69; 27.5%), B2 (6/69; 8.7%), C (6/69; 8.7%), E (6/69; 8.7%), and F (6/69; 8.7%). This result was consistent with a previous study about fecal ESBL/AmpC *E. coli* isolated from healthy Labrador retrievers [19] in which group B1 was the most common phylo-group detected (77/187; 41.2%), followed by C (39/187; 20.9%), B2 (31/187; 16.6%), A (16/187; 8.6%) [19]. Our prevalence of ESBL/AmpC-producing *E. coli* (25.9%, 69/266) is consistent with Belas et al. [5] (25.2%, 33/131), Benavides et al. [20] (24.4%, 20/82) and Aslantaş and Yilmaz [21] (22.2%, 95/428) while appears higher than van den Bunt et al. [22] (10.6%, 59/555) and Wedley et al. [17] (7.1% of AmpC and 1.9% of ESBL over 581 canine fecal samples). Although the results of our study appear consistent with what already reported in literature, the lack of a standardized diagnostic method makes difficult the comparison of results emerged from different studies. [23]. In particular, some differences in the diagnostic approach should be considered such as the use of commercial or in-house agar or cephalosporin chosen as supplement as well as its amount, which may vary between study protocols (e.g. the concentration of KB disk or the dilution of 1 μg/mL or 2 μg/mL in the supplement).

In this study, the higher prevalence of ESBL/AmpC-producing *E. coli* was recorded in domestic dogs that frequent extra-urban environments identifying natural environments as a risk factor. Beside the role of “conductor” in the transmission of pathogenic strains/resistance genes played by environment [24], our result is supported by van den Bunt et al. [22] and Benavides et al. [20] who defined agricultural settings/natural ecosystems as risk factors for ESBL *E. coli* in dogs assuming a potential exposure to these pathogenic bacteria or AMR genes from other animals (wild or domestic) or from their fecal material. Previous studies reported that another risk factor for the increase in prevalence of these pathogenic bacteria is the consumption of raw meat, even poultry, an event that would be more likely to occur for a hunting dog [17,19,21]. However, in the present study, considering only extra urban dogs, there was no difference in prevalence of ESBL/AmpC *E. coli* between hunting and not hunting dogs. Thus, the hunting activity cannot be identified as a risk factor in our study. In any case, the high prevalence of AMR bacteria recorded in domestic dogs poses attention to the frequent contacts that usually occur between dogs and humans, particularly owners living in close contacts. Indeed, domestic dogs can play a key role as conductor or intermediary host between environment and humans, and vice-versa. Thus, in addition to risks related to the food chain, those of AMR and of potential antimicrobial treatment failures [25] related to companion animals, particularly dogs, should not be underestimated.

The *bla*_CTX-M_ was the most frequent (79.7%, 55/69) among AMR genes, as reported previously [5,20]. The findings of TEM-1 (24/25) and SHV-12 (4/4) as the most frequent variants of *bla*_TEM_ and *bla*_SHV_ are consistent with previous studies [26,27,28]. Although *bla*_CMY_ would appear not to have such a high prevalence (13.0%, 9/69), the negative effects of this AMR gene seems to emerge. Indeed, besides the 100% of resistances to cefotaxime (69/69), mainly induced by *bla*_CTX-M_ [29], ESBL/AmpC *E. coli* of this study expressing *bla*_CMY_ were positively associated to resistance to FOX, F/C and T/C. This result is supported by previous studies [29,30] showing that AmpC enzymes are not inhibited by β-lactamase inhibitors, such as clavulanic acid. Moreover, the negative effects of *bla*_CMY_ emerged also in the increased number of antimicrobials an isolate expressing this gene was resistant to. The choice was to investigate these four AMR genes due to their most frequently detection when ESBL/AmpC *E. coli* was isolated not only in dogs [5,17,20,21,22] but also in other animal species [31,32,33] as well as in humans [34,35,36]. Concerning our study area, the only data currently available about the presence of these resistant genes in ESBL/AmpC *E. coli* are those from a study on wild boar and even in that wild species *bla*_CTX-M_ was the most frequently detected with a prevalence of 12.3% (185/1504) followed by *bla*_TEM_ (6.98%, 105/1504), *bla*_CMY_ (0.86%, 13/1504) and *bla*_SHV_ (0.47%, 7/1504) [37]. In this regard, focusing on the spread of AMR genes in humans, during 1990s, the most ESBLs were mutants of the classical TEM-1, -2 and SHV-1 enzymes [38] and *bla*_TEM_ was the most frequent isolated among nosocomial strains [39]. From the 2000s the *bla*_CTX-M_ genes in ESBL *E. coli* became the dominant enzymes in human populations worldwide [40] and particularly in Italy with recorded prevalence of CTX-M-type between 19.7% (115/583) and 94% (232/247) [36,38,41,42,43]. The findings of 7 ESBL/AmpC-producing *E. coli* resistant to ertapenem and one isolate resistant to both ertapenem and meropenem is consistent to previous studies about *E. coli* and ESBL-producing *E. coli* isolated from dogs’ feces [44,45,46,47]. Although to date, carbapenem-resistances are still uncommon in non-human sources and have been recognized only sporadically in domestic animals [44], the fact that carbapenems remain first-line agents for treatments of ESBL/AmpC *E. coli* infections [48] leads to their extensive use that resulted in increasing resistances [49]. The fact that four of these ESBL/AmpC *E. coli* that showed resistance to carbapenems expressed *bla*_CTX-M_ (*n* = 2) and *bla*_CTX-M +_ *bla*_TEM_ (*n* = 2) while the other four carried *bla*_CMY_ (*n* = 3) and *bla*_CMY +_ *bla*_TEM_ (*n* = 1) leads to further molecular investigations about carbapenemase resistance genes since AmpC-producing isolates are susceptible to carbapenems [30] and that a co-occurrence of AMR genes to cephalosporins and carbapenems was reported [46]. In any case, this spread of carbapenemase resistance in domestic dogs, although still limited, combined with the synanthropic role of domestic dogs, should be kept under control and further investigations would be useful in defining the level of spread of these bacteria in humans living in close contact in order to define whether these recorded patterns are of animal or human origin.

## 4. Materials and Methods

### 4.1. Sampling

During 2018 and 2019, a total of 266 fecal samples were collected for research purposes from as many healthy dogs of two provinces of North Eastern Italy. A convenience sampling was carried out in collaboration with three veterinarians. Samples were conferred to the Diagnostic Department of IZSLER (Istituto Zooprofilattico Sperimentale della Lombardia e dell’Emilia Romagna) in Brescia (Italy) for the subsequent diagnostic analysis. During sampling, anamnestic information was registered for the research purposes. For 243 individuals the age was available and dogs were grouped into age class 1 “puppies” (≤1 year old; *n* = 57), age class 2 “adults” (2–10 years old; *n* = 161) and age class 3 “old” (11–15 years old; *n* = 25). A total of 77 dogs lived in the city and frequented exclusively urban environment (e.g., green city areas and urban parks). While a total of 189 dogs lived in the countryside and attended extra-urban environments (agricultural and rural areas of agro-sylvo-pastoral interest also near to small creeks). Out of the 189 extra-urban dogs, 131 were hunting dogs. None of the sampled dogs had received any antibiotic treatment in the last six months.

### 4.2. Isolation and Identification of ESBL/AmpC E. coli

The identification of β-lactamase-producing *E. coli* was performed through a double synergy diagnostic method. Specifically, 1 g of feces has been diluted in 9 mL (1:10 dilution) of brain heart infusion (BHI) broth supplemented with 1 mg/L cefotaxime for a pre-enrichment phase. After an overnight incubation, a drop of the BHI broth was used to inoculate MacConkey agar supplemented with 1 mg/L cefotaxime [49]. Positive growths were identified as pink to dark-pink colonies (lactose +). A single bacterial colony from each phenotype-positive sample was resuspended in 250 μL of DNase-Rnase free water and DNA was extracted by lysis-boiling (98 °C for 10 min) for further molecular characterization. Identification of *E. coli* was conducted by a PCR phylogenetic group analysis according to Clermont et al. [50].

### 4.3. Analysis of Resistance Genes

The detection of resistance genes was performed through a multiplex PCR using specific primers [51] for *bla*_CTX-M_, *bla*_TEM_ and *bla*_SHV_ and according to Rehman et al. [52] for *bla*_CMY_. The PCR amplification was carried out using DreamTaq Green PCR master mix (Thermo scientific, Waltham, MA, USA). Briefly, 25 μL of reaction mix contained 12.5 μL DreamTaq Green PCR master mix (2X), 0.25 μL (10 μM) each of forward and reverse primers of *bla*_TEM_ and *bla*_SHV_, 0.75 µL (10 μM) each of forward and reverse primers of *bla*_CTX-M_ and *bla*_CMY_, 2 μL of template DNA and 6.5 μL of nuclease-free water (NFW). The amplification parameters were set as follows: 95 °C for 3 min, followed by 30 cycles at 95 °C for 30 s, 59 °C for 30 s, and 72 °C for 1 min, and a final extension phase of 72 °C for 7 min. All amplicons found to be positive for *bla*_TEM_ and *bla*_SHV_ were sequenced [53]. Sequences were deposited in NCBI GenBank with accession numbers from OK037506 to OK037530 for *bla*_TEM_ and from OK037531 to OK037534 for *bla*_SHV_.

### 4.4. Antimicrobial Susceptibility Testing

All phenotype-positive *E. coli* isolates were subjected to antimicrobial susceptibility testing. Minimum inhibitory concentrations (MICs) were determined by broth microdilution using commercial plates (EUVSEC2 Sensititre^TM^ plates, Trek diagnostics, Thermo Scientific^®^) (Table 6). Strains were classified as susceptible or resistant based on epidemiological cut-off values (ECOFFs) recommended by the European Committee on Antimicrobial Susceptibility Testing (EUCAST, www.eucast.org accessed on 8 September 2021), as described in the Decision 2013/652/EU [54,55] (Table 6).

### 4.5. Statistical Analysis

Risk factors associated to ESBL/AmpC *E*. *coli* infection in dogs were assessed through a logistic regression model, analysing the effect of age class (1, 2 or 3) and type of frequented environment (urban/extra urban) on the infection status (infected/not infected). Additionally, on the subset of dogs that frequented extra-urban environments, differences in ESBL/AmpC *E*. *coli* prevalence between hunting and non-hunting dogs were assessed through a chi-square test. Through another set of logistic regressions, we analysed whether the same variables had any effect on the presence (present/absent) of the four resistance genes detected by PCR in positive samples. On the positive samples, we also investigated the association among ESBL/AmpC *E. coli* phylogenetic groups and the presence of the four resistance genes by Fisher’s exact tests. Then, by a set of nine logistic regressions, we examined the relationship between resistance to the nine antimicrobials tested by MIC (resistant/susceptible) and the presence of the four genes, including the phylogenetic group as a covariate. Firth’s penalized maximum likelihood estimation method was applied to reduce the bias for rare events.

Variation in the number of antimicrobials each isolate was resistant to (i.e., from 0 to 9) was analysed through a Poisson regression, including phylogenetic group and the presence of the four genes as explanatory variables. Finally, we examined the effect of these same variables on multidrug resistance (MDR), according to the definition reported by Sweeney et al. [57].

All the analyses were carried out using SAS/STAT 9.4 software (Copyright © 2021, SAS Institute Inc., Cary, NC, USA).

## 5. Conclusions

The high prevalence of ESBL/AmpC-producing *E. coli* recorded in this study shows the role that domestic dogs may play in maintaining and transmitting these infections, even potentially to close-contact humans. Natural environments represent a risk factor for the spread of ESBL/AmpC-producing *E. coli* strains likely because they can act as “collectors of species” being frequented by many domestic and wild animals that can contribute to the cycle and the spread of these strains.

In addition to the high resistance to third and fourth generation cephalosporins, in a few isolates resistance to carbapenems has also been found and this point leads to new studies. Indeed, both ESBL/AmpC- and carbapenemase-producing *E. coli* should be further monitored, mostly in dogs that live in such close contact with humans. On one hand, molecular analysis of AMR genes should include carbapenemases genes in order to assess their spread and the potential relation to the recorded microbiological resistances of strains. On the other hand, given the recorded molecular and microbiological resistances, the study should carry on including information about the use of antimicrobials in small domestic animals in order to evaluate the potential relations between antimicrobials used and the onset/occurrence of resistances or their modifications during time.

## Figures and Tables

**Table 1:** Occurrence of AMR resistance genes in ESBL/AmpC *E. coli* isolated from dogs’ faecal samples (*n* = 69) and their association with phylogenetic groups.

Genes	%	% by Phylogenetic Group							*p*-Value
		A (*n* = 19)	B1 (*n* = 21)	B2 (*n* = 6)	C (*n* = 6)	D (*n* = 5)	E (*n* = 6)	F (*n* = 6)	
*bla*_CTX-M_	79.7	84.2	66.7	83.3	100	80	66.7	100	0.61
*bla*_TEM_	47.8	57.9	33.3	33.3	83.3	0	83.3	50	0.03
*bla*_CMY_	13	10.5	19.0	16.7	0	20	16.7	0	0.65
*bla*_SHV_	5.8	10.5	9.5	0	0	0	0	0	1.00

**Table 2:** Combination of AMR genes found in ESBL/AmpC *E. coli* isolated from dogs’ faecal samples (*n* = 69).

Genes Combination	*n*	%	95% CI
*bla*_CTX-M_	27	39.1	27.3–50.9
*bla*_CTX-M_ + *bla*_TEM_	25	36.2	24.6–47.9
*bla*_TEM_	4	5.8	0.1–11.4
*bla*_CMY_	4	5.8	0.1–11.4
*bla*_SHV_	3	4.3	0–9.3
*bla*_CTX-M_ + *bla*_CMY_	2	2.9	0–7.0
*bla*_TEM_ + *bla*_CMY_	2	2.9	0–7.0
*bla*_CTX-M_ + *bla*_TEM_ + *bla*_SHV_	1	1.4	0–4.3

**Table 3:** Minimum inhibitory concentrations (MICs) of ESBL/AmpC *E. coli* isolated from dogs’ faecal samples (*n* = 69). Cefepime (FEP), Cefotaxime (FOT), Cefotaxime/clavulanic acid (F/C), Cefoxitin (FOX), Ceftazidime (TAZ), Ceftazidime/clavulanic acid (T/C), Ertapenem (ETP), Imipenem (IMI), Meropenem (MERO), Temocillin (TRM).

	Distribution (%) of MIC Values (mg/L)													
Antimicrobial	0.015	0.03	0.06	0.125	0.25	0.5	1	2	4	8	16	32	64	128
FEP			1.4	5.7	7.1	4.3	4.3	12.9	40.0	22.9	1.4			
FOT					1.4			5.7	5.7	7.1	32.9	20.0	27.1	
F/C			60.0	20.0	1.4	1.4	1.4	4.3	1.4	4.3	2.9	1.4	1.4	
FOX								11.4	52.9	15.7	8.6	1.4	10.0	
TAZ					1.4	10.0	15.7	21.4	10.0	20.0	12.9	4.3	1.4	2.9
T/C				54.3	24.3	4.3		1.4	2.9	4.3	4.3		2.9	1.4
ETP	61.4	27.1	7.1	1.4	1.4			1.4						
IMI				98.6		1.4								
MERO		97.1	1.4						1.4					
TRM						1.4		1.4	18.6	65.7	10.0	1.4		1.4

**Table 4:** Resistance to antimicrobials in ESBL/AmpC *E. coli* isolated from dogs’ feaces (*n* = 69): percentage of isolates resistant to each of the ten antimicrobials tested by MIC and occurrence of specific genes. Cefepime (FEP), cefotaxime (FOT), cefotaxime/clavulanic acid (F/C), cefoxitin (FOX), ceftazidime (TAZ), ceftazidime/clavulanic acid (T/C), ertapenem (ETP), imipenem (IMI), meropenem (MERO), temocillin (TRM).

Antimicrobial	% of Resistant Isolates	% of Resistant Isolates with Associated Genes			
		*bla*_CTX-M_	*bla*_SHV_	*bla*_CMY_	*bla*_TEM_
FEP (*n* = 60)	86.9	85	3.3	8.3	50.0
FOT (*n* = 69)	100	79.7	5.8	13.0	47.8
F/C (*n* = 13)	18.8	46.1	0	61.5	30.8
FOX (*n* = 14)	20.3	42.8	0	57.1	35.7
TAZ (*n* = 62)	89.9	79.0	6.4	14.5	46.8
T/C (*n* = 12)	17.4	41.7	0	66.7	25.0
ETP (*n* = 8)	11.6	50.0	0	50.0	37.5
IMI	0	0	0	0	0
MERO (*n* = 1)	1.4	100	0	0	0
TRM (*n* = 2)	2.9	50	0	50	0

**Table 5:** Logistic regression models showing variation in the probability for an ESBL/AmpC *E. coli* isolate (*n* = 69) of being resistant to specific antimicrobials in relation to the presence of selected resistance genes. Only models with at least one significant explanatory variable are reported. Cefoxitin (FOX), cefotaxime/clavulanic acid (F/C), and ceftazidime/clavulanic acid (T/C).

Antimicrobial	Gene	Parameter Estimate ± SE	X^2^_1_	*p*-Value
FOX	*bla*_CMY_	1.15 ± 0.58	3.87	0.049
	*bla*_CTX-M_	−1.15 ± 0.60	3.73	0.054
	*bla*_TEM_	−0.57 ± 0.48	1.37	0.24
	*bla*_SHV_	−1.25 ± 1.08	1.34	0.25
F/C	*bla*_CMY_	1.39 ± 0.61	5.19	0.023
	*bla*_CTX-M_	−0.75 ± 0.64	1.38	0.24
	*bla*_TEM_	−0.56 ± 0.48	1.39	0.24
	*bla*_SHV_	−0.81 ± 1.08	0.56	0.45
T/C	*bla*_CMY_	1.33 ± 0.61	4.75	0.029
	*bla*_CTX-M_	−1.30 ± 0.85	2.33	0.13
	*bla*_TEM_	−1.13 ± 0.73	2.40	0.12
	*bla*_SHV_	−1.36 ± 1.25	1.19	0.28

**Table 6:** List of antimicrobials (EUVSEC2 Sensititre^TM^ plates), the related cut-off values (ECOFFs, EUCAST, www.eucast.org accessed on 8 September 2021) and the range of minimum inhibitory concentration (MIC, mg/L) according to CLSI [56].

Antimicrobial	Interpretative Thresholds of AMR (mg/L) ECOFF (R > mg/L) ^1^	Range of Concentrations (mg/L) (N° of Wells in Brackets)
Cefepime (FEP)	0.25	0.06–32 (10)
Cefotaxime (FOT)	0.25	0.25–64 (9)
Cefotaxime/clavulanic acid (F/C)	0.25	0.06–64 (11)
Cefoxitin (FOX)	8	0.5–64 (8)
Ceftazidime (TAZ)	0.5	0.25–128 (10)
Ceftazidime/clavulanic acid (T/C)	0.5	0.125–128 (11)
Ertapenem (ETP)	0.03	0.015–2 (8)
Imipenem (IMI)	0.5	0.12–16 (8)
Meropenem (MERO)	0.06	0.03–16 (10)
Temocillin (TRM)	16	0.5–128 (9)

^1^ EUCAST Clinical Breakpoint Tables. https://eucast.org/mic_distributions_and_ecoffs/, https://www.eucast.org/mic_and_zone_distributions_and_ecoffs/new_and_revised_ecoffs/ (accessed on 8 September 2021).

## Data Availability

The raw data supporting the conclusions of this article will be made available by the authors, without undue reservation.
